# Methyl 3-oxo-2,3-dihydro-1,2-benzothia­zole-2-acetate 1,1-dioxide

**DOI:** 10.1107/S1600536808009951

**Published:** 2008-04-16

**Authors:** Waseeq Ahmad Siddiqui, Saeed Ahmad, Hamid Latif Siddiqui, Masood Parvez, Rehana Rashid

**Affiliations:** a Department of Chemistry, University of Sargodha, Sargodha, Pakistan; b Department of Chemistry, University of Science and Technology, Bannu, Pakistan; c Institute of Chemistry, University of the Punjab, Lahore, Pakistan; d Department of Chemistry, University of Calgary, 2500 University Drive NW, Calgary, Alberta, Canada T2N 1N4; e Department of Chemistry, University of Balochistan, Quetta, Pakistan

## Abstract

The title mol­ecule, C_10_H_9_NO_5_S, is composed of two essentially planar units with a dihedral angle of 89.16 (6)° between them. In the crystal structure, there are weak inter­molecular C—H⋯O inter­actions resulting in dimeric pairs of mol­ecules about inversion centres and chains of mol­ecules extended along the *a* and *c* axes, thus stabilizing the structure. In addition, benzothia­zole rings lying parallel to each other with centroid–centroid distances of 3.679 (2) and 3.999 (2) Å indicate the existence of π–π stacking inter­actions.

## Related literature

For related literature, see: Kapui *et al.* (2003 ▶); Masashi *et al.* (1999 ▶); Manjarrez *et al.* (1996 ▶); Siddiqui, Ahmad, Khan, Siddiqui & Parvez (2007 ▶); Siddiqui, Ahmad, Khan, Siddiqui & Weaver (2007 ▶); Siddiqui, Ahmad, Siddiqui *et al.* (2007 ▶); Siddiqui *et al.* (2008 ▶); Xu *et al.* (2005 ▶, 2006 ▶).
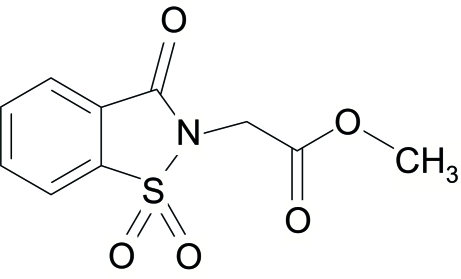


## Experimental

### 

#### Crystal data

C_10_H_9_NO_5_S
*M* *_r_* = 255.24
Triclinic, 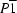

*a* = 7.765 (3) Å
*b* = 8.496 (3) Å
*c* = 8.776 (4) Å
α = 104.39 (2)°
β = 100.58 (2)°
γ = 94.30 (2)°
*V* = 546.8 (4) Å^3^
*Z* = 2
Mo *K*α radiation
μ = 0.30 mm^−1^
*T* = 173 (2) K
0.16 × 0.10 × 0.08 mm

#### Data collection

Nonius KappaCCD diffractometer
Absorption correction: multi-scan (*SORTAV*; Blessing, 1997 ▶) *T*_min_ = 0.953, *T*_max_ = 0.976
4654 measured reflections
2468 independent reflections
2040 reflections with *I* > 2σ(*I*)
*R*_int_ = 0.024

#### Refinement

*R*[*F* ^2^ > 2σ(*F* ^2^)] = 0.040
*wR*(*F* ^2^) = 0.111
*S* = 1.03
2468 reflections
155 parameters
H-atom parameters constrained
Δρ_max_ = 0.46 e Å^−3^
Δρ_min_ = −0.43 e Å^−3^

### 

Data collection: *COLLECT* (Hooft, 1998 ▶); cell refinement: *DENZO* (Otwinowski & Minor, 1997 ▶); data reduction: *SCALEPACK* (Otwinowski & Minor, 1997 ▶); program(s) used to solve structure: *SAPI91* (Fan, 1991 ▶); program(s) used to refine structure: *SHELXL97* (Sheldrick, 2008 ▶); molecular graphics: *ORTEP-3* (Farrugia, 1997 ▶); software used to prepare material for publication: *SHELXL97*.

## Supplementary Material

Crystal structure: contains datablocks global, I. DOI: 10.1107/S1600536808009951/lh2607sup1.cif

Structure factors: contains datablocks I. DOI: 10.1107/S1600536808009951/lh2607Isup2.hkl

Additional supplementary materials:  crystallographic information; 3D view; checkCIF report

## Figures and Tables

**Table 1:** Hydrogen-bond geometry (Å, °)

*D*—H⋯*A*	*D*—H	H⋯*A*	*D*⋯*A*	*D*—H⋯*A*
C2—H2⋯O5^i^	0.95	2.53	3.435 (3)	160
C4—H4⋯O4^ii^	0.95	2.54	3.209 (3)	128
C8—H8*A*⋯O2^iii^	0.99	2.49	3.435 (3)	159
C10—H10*C*⋯O1^iv^	0.98	2.47	3.431 (3)	167

Symmetry codes: (i) 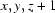; (ii) 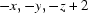; (iii) 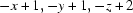; (iv) 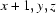.

**Fig. 1.**
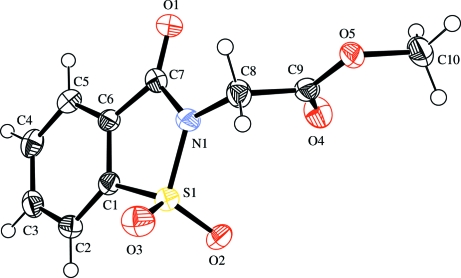
ORTEP-3 (Farrugia, 1997) drawing of (I) with displacement ellipsoids plotted at 50% probability level.

**Fig. 2.**
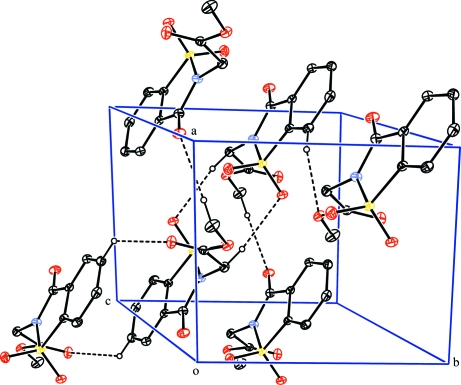
Unit cell packing of (I) showing C—H···O interactions represented by dashed lines; H-atoms not involved in H-bonds have been omitted.

**Fig. 3.**
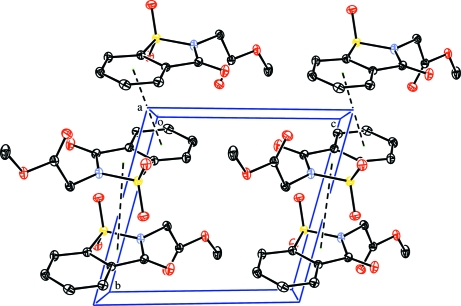
Unit cell packing of (I) showing π-π stacking interactions represented by dashed lines; H-atoms have been omitted for clarity.

## supplementary crystallographic information

### Comment

Saccharin derivatives are considered to be the most potent orally active human leucocyte elastase (HLE) inhibitors for the treatment of chronic obstructive pulmonary disease (COPD), acute respiratory distress syndrome (ARDS), cystic fibrosis, asthma and other inflammatory diseases (Kapui *et al.*, 2003). Various biologically important saccharin skeletons and their *N*-alkyl derivatives were efficiently prepared (Xu *et al.*, 2006) by chromium oxide-catalyzed oxidation of *N*-alkyl(*o*-methyl)arenesulfonamides in acetonitrile besides the already developed methodology utilizing irradiation techniques for similar type of conversions (Masashi *et al.*, 1999). In continuation to our research on benzene, 1,2-benzothiazine 1,1-dioxide and saccharin derivatives (Siddiqui *et al.*, 2008; Siddiqui, Ahmad, Khan, Siddiqui & Weaver, 2007), we report herein the crystal structure of the title compound, (I).

The structure of (I) is composed of an essentially planar moiety, S1/N1/O1/C1—C7 with maximum deviations from the least-square planes being: O1 = -0.0540 (12) and N1 = 0.0540 (13) Å, and an approximately planar moiety C8—C10/O4/O5 with maximum deviation of 0.0766 (13) Å for C8. The two moieties are oriented with a dihedral angle of 89.16 (6)° between their least-squares planes. The structure is stabilized by four rather weak intermolecular interactions of the type C—H···O (Fig. 2 and Table 1). Of these interactions, C4—H4···O5 and C8—H8A···O2 H-bonds result in dimeric pairs of (I) while C10—H10···O1 and C2—H2···O5 result in chains of molecules extended along the a- and c-axes, respectively. The benzothiazole rings in (I) lie parallel to each other about the origin with the shortest distance between the centroids of the benzene rings of the adjacent molecules is 3.679 (2) Å which indicates the existence of π-π stacking interactions. The thiazoline rings located about inversion centers in the middle of the *b* axis (at 0, 1/2, 0) also show π-π interaction with centroids of these rings separated by 3.999 (2) Å (Fig. 3). The molecular dimensions in (I) are in agreement with the corresponding dimensions reported in similar structures (Xu *et al.*, 2005; Siddiqui, Ahmad, Khan, Siddiqui & Parvez, 2007; Siddiqui, Ahmad, Siddiqui *et al.*, 2007; Siddiqui *et al.*, 2008).

### Experimental

The compound (I) was prepared following the prcedures reported earlier (Manjarrez *et al.*, 1996). Crystals suitable for X-ray crystallography were grown from a solution of CH_3_OH by slow evaporation at 313 K.

### Refinement

H-atoms were included in the refinements at geometrically idealized positions with aryl, methylene and methyl C—H distances 0.95, 0.99 and 0.98 Å, respectively, and *U*_iso_ = 1.2 times *U*_eq_ of the atoms to which they were bonded. The final difference map was free of any chemically significant features.

### Crystal data

**Table:**

C_10_H_9_NO_5_S	*Z* = 2
*M**_r_* = 255.24	*F*_000_ = 264
Triclinic, *P*1	*D*_x_ = 1.550 Mg m^−^^3^
Hall symbol: -P 1	Mo *K*α radiation λ = 0.71073 Å
*a* = 7.765 (3) Å	Cell parameters from 4654 reflections
*b* = 8.496 (3) Å	θ = 3.9–27.4º
*c* = 8.776 (4) Å	µ = 0.31 mm^−^^1^
α = 104.39 (2)º	*T* = 173 (2) K
β = 100.58 (2)º	Block, colourless
γ = 94.30 (2)º	0.16 × 0.10 × 0.08 mm
*V* = 546.8 (4) Å^3^	

### Data collection

**Table:**

Nonius KappaCCD diffractometer	2468 independent reflections
Radiation source: fine-focus sealed tube	2040 reflections with *I* > 2σ(*I*)
Monochromator: graphite	*R*_int_ = 0.024
*T* = 173(2) K	θ_max_ = 27.4º
ω and φ scans	θ_min_ = 3.9º
Absorption correction: multi-scan(SORTAV; Blessing, 1997)	*h* = −10→9
*T*_min_ = 0.953, *T*_max_ = 0.976	*k* = −11→10
4654 measured reflections	*l* = −11→11

### Refinement

**Table:**

Refinement on *F*^2^	Secondary atom site location: difference Fourier map
Least-squares matrix: full	Hydrogen site location: inferred from neighbouring sites
*R*[*F*^2^ > 2σ(*F*^2^)] = 0.040	H-atom parameters constrained
*wR*(*F*^2^) = 0.111	*w* = 1/[σ^2^(*F*_o_^2^) + (0.056*P*)^2^ + 0.29*P*] where *P* = (*F*_o_^2^ + 2*F*_c_^2^)/3
*S* = 1.03	(Δ/σ)_max_ < 0.001
2468 reflections	Δρ_max_ = 0.46 e Å^−^^3^
155 parameters	Δρ_min_ = −0.43 e Å^−^^3^
Primary atom site location: structure-invariant direct methods	Extinction correction: none

### Special details

**Table:**

Geometry. All e.s.d.'s (except the e.s.d. in the dihedral angle between two l.s. planes) are estimated using the full covariance matrix. The cell e.s.d.'s are taken into account individually in the estimation of e.s.d.'s in distances, angles and torsion angles; correlations between e.s.d.'s in cell parameters are only used when they are defined by crystal symmetry. An approximate (isotropic) treatment of cell e.s.d.'s is used for estimating e.s.d.'s involving l.s. planes.
Refinement. Refinement of *F*^2^ against ALL reflections. The weighted *R*-factor *wR* and goodness of fit *S* are based on *F*^2^, conventional *R*-factors *R* are based on *F*, with *F* set to zero for negative *F*^2^. The threshold expression of *F*^2^ > σ(*F*^2^) is used only for calculating *R*-factors(gt) *etc*. and is not relevant to the choice of reflections for refinement. *R*-factors based on *F*^2^ are statistically about twice as large as those based on *F*, and *R*- factors based on ALL data will be even larger.

### Fractional atomic coordinates and isotropic or equivalent isotropic displacement parameters (Å^2^)

**Table:**

	*x*	*y*	*z*	*U*_iso_*/*U*_eq_	
S1	0.25284 (6)	0.37128 (6)	1.06522 (6)	0.02602 (16)	
O1	−0.07412 (18)	0.18372 (19)	0.67891 (17)	0.0354 (3)	
O2	0.40672 (17)	0.29152 (17)	1.08988 (17)	0.0320 (3)	
O3	0.2727 (2)	0.54422 (17)	1.13620 (18)	0.0372 (4)	
O4	0.3882 (2)	0.14876 (17)	0.70088 (18)	0.0369 (4)	
O5	0.41016 (18)	0.33528 (17)	0.56027 (16)	0.0312 (3)	
N1	0.1635 (2)	0.3311 (2)	0.87005 (18)	0.0275 (4)	
C1	0.0687 (2)	0.2676 (2)	1.1032 (2)	0.0243 (4)	
C2	0.0451 (3)	0.2516 (2)	1.2517 (2)	0.0303 (4)	
H2	0.1321	0.2987	1.3471	0.036*	
C3	−0.1118 (3)	0.1633 (3)	1.2543 (3)	0.0325 (4)	
H3	−0.1321	0.1482	1.3535	0.039*	
C4	−0.2397 (3)	0.0968 (2)	1.1148 (3)	0.0329 (5)	
H4	−0.3467	0.0384	1.1202	0.040*	
C5	−0.2128 (3)	0.1147 (2)	0.9669 (2)	0.0297 (4)	
H5	−0.3005	0.0698	0.8716	0.036*	
C6	−0.0558 (2)	0.1990 (2)	0.9619 (2)	0.0245 (4)	
C7	−0.0003 (2)	0.2326 (2)	0.8179 (2)	0.0263 (4)	
C8	0.2499 (3)	0.3960 (2)	0.7616 (2)	0.0292 (4)	
H8A	0.3291	0.4972	0.8238	0.035*	
H8B	0.1593	0.4256	0.6819	0.035*	
C9	0.3566 (2)	0.2757 (2)	0.6733 (2)	0.0268 (4)	
C10	0.5248 (3)	0.2391 (3)	0.4726 (3)	0.0435 (6)	
H10A	0.5553	0.2910	0.3914	0.052*	
H10B	0.4638	0.1284	0.4195	0.052*	
H10C	0.6329	0.2329	0.5475	0.052*	

### Atomic displacement parameters (Å^2^)

**Table:**

	*U*^11^	*U*^22^	*U*^33^	*U*^12^	*U*^13^	*U*^23^
S1	0.0241 (3)	0.0292 (3)	0.0245 (3)	0.00447 (18)	0.00385 (18)	0.00737 (18)
O1	0.0311 (7)	0.0495 (9)	0.0236 (7)	0.0080 (6)	0.0024 (6)	0.0076 (6)
O2	0.0225 (7)	0.0398 (8)	0.0339 (8)	0.0068 (6)	0.0027 (6)	0.0117 (6)
O3	0.0426 (9)	0.0266 (7)	0.0379 (8)	0.0026 (6)	0.0035 (7)	0.0045 (6)
O4	0.0472 (9)	0.0316 (8)	0.0378 (8)	0.0101 (7)	0.0158 (7)	0.0136 (6)
O5	0.0308 (7)	0.0441 (8)	0.0252 (7)	0.0126 (6)	0.0102 (6)	0.0158 (6)
N1	0.0242 (8)	0.0376 (9)	0.0217 (8)	0.0022 (7)	0.0048 (6)	0.0102 (7)
C1	0.0226 (9)	0.0270 (9)	0.0251 (9)	0.0069 (7)	0.0061 (7)	0.0083 (7)
C2	0.0307 (10)	0.0375 (11)	0.0241 (10)	0.0094 (8)	0.0053 (8)	0.0095 (8)
C3	0.0377 (11)	0.0375 (11)	0.0309 (11)	0.0155 (9)	0.0161 (9)	0.0151 (8)
C4	0.0269 (10)	0.0328 (10)	0.0452 (12)	0.0065 (8)	0.0137 (9)	0.0164 (9)
C5	0.0245 (9)	0.0314 (10)	0.0328 (10)	0.0046 (8)	0.0035 (8)	0.0095 (8)
C6	0.0252 (9)	0.0248 (9)	0.0240 (9)	0.0086 (7)	0.0042 (7)	0.0066 (7)
C7	0.0261 (9)	0.0288 (9)	0.0237 (10)	0.0051 (7)	0.0073 (7)	0.0042 (7)
C8	0.0305 (10)	0.0329 (10)	0.0297 (10)	0.0075 (8)	0.0115 (8)	0.0135 (8)
C9	0.0230 (9)	0.0325 (10)	0.0243 (9)	0.0000 (7)	0.0032 (7)	0.0090 (8)
C10	0.0436 (13)	0.0656 (15)	0.0302 (11)	0.0250 (11)	0.0173 (10)	0.0169 (10)

### Geometric parameters (Å, °)

**Table:**

S1—O2	1.4258 (15)	C3—C4	1.389 (3)
S1—O3	1.4306 (15)	C3—H3	0.9500
S1—N1	1.6640 (18)	C4—C5	1.395 (3)
S1—C1	1.7504 (19)	C4—H4	0.9500
O1—C7	1.202 (2)	C5—C6	1.380 (3)
O4—C9	1.196 (2)	C5—H5	0.9500
O5—C9	1.335 (2)	C6—C7	1.492 (3)
O5—C10	1.449 (2)	C8—C9	1.517 (3)
N1—C7	1.402 (3)	C8—H8A	0.9900
N1—C8	1.449 (2)	C8—H8B	0.9900
C1—C2	1.387 (3)	C10—H10A	0.9800
C1—C6	1.388 (3)	C10—H10B	0.9800
C2—C3	1.389 (3)	C10—H10C	0.9800
C2—H2	0.9500		
			
O2—S1—O3	116.71 (9)	C6—C5—H5	120.7
O2—S1—N1	110.73 (9)	C4—C5—H5	120.7
O3—S1—N1	109.35 (9)	C5—C6—C1	119.81 (18)
O2—S1—C1	112.26 (9)	C5—C6—C7	127.38 (17)
O3—S1—C1	112.83 (9)	C1—C6—C7	112.78 (17)
N1—S1—C1	92.26 (9)	O1—C7—N1	123.33 (18)
C9—O5—C10	115.33 (16)	O1—C7—C6	128.71 (18)
C7—N1—C8	122.31 (16)	N1—C7—C6	107.93 (16)
C7—N1—S1	116.00 (13)	N1—C8—C9	112.82 (16)
C8—N1—S1	121.68 (13)	N1—C8—H8A	109.0
C2—C1—C6	122.73 (18)	C9—C8—H8A	109.0
C2—C1—S1	126.37 (15)	N1—C8—H8B	109.0
C6—C1—S1	110.90 (14)	C9—C8—H8B	109.0
C1—C2—C3	116.80 (19)	H8A—C8—H8B	107.8
C1—C2—H2	121.6	O4—C9—O5	125.61 (18)
C3—C2—H2	121.6	O4—C9—C8	125.59 (18)
C2—C3—C4	121.34 (19)	O5—C9—C8	108.80 (16)
C2—C3—H3	119.3	O5—C10—H10A	109.5
C4—C3—H3	119.3	O5—C10—H10B	109.5
C3—C4—C5	120.73 (19)	H10A—C10—H10B	109.5
C3—C4—H4	119.6	O5—C10—H10C	109.5
C5—C4—H4	119.6	H10A—C10—H10C	109.5
C6—C5—C4	118.57 (18)	H10B—C10—H10C	109.5
			
O2—S1—N1—C7	111.37 (15)	C2—C1—C6—C5	1.8 (3)
O3—S1—N1—C7	−118.66 (15)	S1—C1—C6—C5	−178.65 (14)
C1—S1—N1—C7	−3.48 (15)	C2—C1—C6—C7	−179.84 (17)
O2—S1—N1—C8	−69.24 (17)	S1—C1—C6—C7	−0.3 (2)
O3—S1—N1—C8	60.73 (17)	C8—N1—C7—O1	6.2 (3)
C1—S1—N1—C8	175.91 (15)	S1—N1—C7—O1	−174.37 (15)
O2—S1—C1—C2	68.07 (19)	C8—N1—C7—C6	−175.64 (16)
O3—S1—C1—C2	−66.31 (19)	S1—N1—C7—C6	3.7 (2)
N1—S1—C1—C2	−178.43 (18)	C5—C6—C7—O1	−5.9 (3)
O2—S1—C1—C6	−111.44 (14)	C1—C6—C7—O1	175.94 (19)
O3—S1—C1—C6	114.19 (14)	C5—C6—C7—N1	176.14 (18)
N1—S1—C1—C6	2.07 (14)	C1—C6—C7—N1	−2.0 (2)
C6—C1—C2—C3	−0.4 (3)	C7—N1—C8—C9	−84.4 (2)
S1—C1—C2—C3	−179.86 (15)	S1—N1—C8—C9	96.29 (18)
C1—C2—C3—C4	−1.0 (3)	C10—O5—C9—O4	−3.8 (3)
C2—C3—C4—C5	1.0 (3)	C10—O5—C9—C8	175.82 (16)
C3—C4—C5—C6	0.4 (3)	N1—C8—C9—O4	−9.1 (3)
C4—C5—C6—C1	−1.8 (3)	N1—C8—C9—O5	171.21 (15)
C4—C5—C6—C7	−179.84 (17)		

### Hydrogen-bond geometry (Å, °)

**Table:**

*D*—H···*A*	*D*—H	H···*A*	*D*···*A*	*D*—H···*A*
C2—H2···O5^i^	0.95	2.53	3.435 (3)	160
C4—H4···O4^ii^	0.95	2.54	3.209 (3)	128
C8—H8A···O2^iii^	0.99	2.49	3.435 (3)	159
C10—H10C···O1^iv^	0.98	2.47	3.431 (3)	167

Symmetry codes: (i) *x*, *y*, *z*+1; (ii) −*x*, −*y*, −*z*+2; (iii) −*x*+1, −*y*+1, −*z*+2; (iv) *x*+1, *y*, *z*.

## References

[bb1] Blessing, R. H. (1997). *J. Appl. Cryst.* **30**, 421–426.

[bb2] Fan, H.-F. (1991). *SAPI91* Rigaku Corporation, Tokyo, Japan.

[bb3] Farrugia, L. J. (1997). *J. Appl. Cryst.* **30**, 565.

[bb4] Hooft, R. (1998). *COLLECT* Nonius BV, Delft, The Netherlands.

[bb5] Kapui, Z., Varga, M., Urban-Szabo, K., Mikus, E., Szabo, T., Szeredi, J., Finance, O. & Aranyi, P. (2003). *J. Pharmacol. Exp. Ther.* **305**, 1–9. 10.1124/jpet.102.044263 12606659

[bb7] Manjarrez, N., Pérez, H. I., Solís, A. & Luna, H. (1996). *Synth. Commun.* **26**, 585–591.

[bb6] Masashi, K., Hideo, T., Kentaro, Y. & Masataka, Y. (1999). *Tetrahedron*, **55**, 14885–14900.

[bb8] Otwinowski, Z. & Minor, W. (1997). *Methods in Enzymology*, Vol. 276, *Macromolecular Crystallography*, Part A, edited by C. W. Carter Jr & R. M. Sweet, pp. 307–326. New York: Academic Press.

[bb9] Sheldrick, G. M. (2008). *Acta Cryst.* A**64**, 112–122. 10.1107/S0108767307043930 18156677

[bb10] Siddiqui, W. A., Ahmad, S., Khan, I. U., Siddiqui, H. L. & Parvez, M. (2007). *Acta Cryst.* E**63**, o4116.

[bb11] Siddiqui, W. A., Ahmad, S., Khan, I. U., Siddiqui, H. L. & Weaver, G. W. (2007). *Synth. Commun.* **37**, 767–773.

[bb12] Siddiqui, W. A., Ahmad, S., Siddiqui, H. L., Tariq, M. I. & Parvez, M. (2007). *Acta Cryst.* E**63**, o4001.

[bb13] Siddiqui, W. A., Ahmad, S., Tariq, M. I., Siddiqui, H. L. & Parvez, M. (2008). *Acta Cryst.* C**64**, o4–o6. 10.1107/S0108270107059173 18216455

[bb14] Xu, L., Shu, H., Liu, Y., Zhang, S. & Trudell, M. (2006). *Tetrahedron*, **62**, 7902–7910.

[bb15] Xu, L.-Z., Si, G.-D., Li, Z.-F., Yang, S.-H. & Li, K. (2005). *Acta Cryst.* E**61**, o1329–o1330.

